# Calvarial Metastasis of Breast Carcinoma Manifesting as a New Daily Persistent Headache: A Case Report

**DOI:** 10.1002/ccr3.72150

**Published:** 2026-07-23

**Authors:** Neha Lall, Anand Kumar, Deepika Joshi, Pramod Kumar Singh

**Affiliations:** ^1^ Department of Radiotherapy & Radiation Medicine Institute of Medical Sciences, BHU Varanasi Uttar Pradesh India; ^2^ Department of Neurology Institute of Medical Sciences, BHU Varanasi Uttar Pradesh India; ^3^ Department of Radiodiagnosis and Imaging Institute of Medical Sciences, BHU Varanasi Uttar Pradesh India

**Keywords:** breast carcinoma, calvarial metastasis, MRI brain, new daily persistent headache

## Abstract

New‐onset daily persistent headache (NDPH) is a primary headache syndrome characterized by a sudden onset of daily headaches persisting for more than three months in patients with no prior headache history. We report the case of a 49‐year‐old Indian lady who presented to the neurology outpatient clinic with new daily persistent headache. The headache had an insidious onset, described as continuous, holocranial, and dull aching. Visual analog scale (VAS) of pain severity was estimated to be 8/10. Her medical history was significant for carcinoma of the left breast cT3N1(Stage IIIA), for which she had received treatment two and a half years prior and was subsequently declared disease‐free. Although symptomatic treatment for her headache was initiated, she remained refractory to standard therapy. A gadolinium‐enhanced magnetic resonance imaging of the brain revealed large, heterogeneous, enhancing areas within the diploic space of the frontal bone. The patient subsequently received palliative external beam radiotherapy. At the 3‐month follow‐up post radiotherapy, her pain had significantly subsided, with a recorded VAS score of 2/10. In conclusion, patients presenting with acute headaches, particularly in the context of cancer, warrant neuro‐radiological imaging. Timely diagnosis of calvarial metastasis is critical in preventing further disease progression and enhancing the patient's quality of life.

**Key Clinical Messages**

Early neuroimaging is mandatory in cancer survivors presenting with atypical headache. A prompt diagnosis of distant metastasis can help prevent further progression and improve the quality of the patient's life.

**Abbreviations**
AJCC: American Joint Committee on Cancer
CARE: CAse Reports
CECT: Contrast‐enhanced computed tomography
EBRT: External beam radiotherapy
ER +: Estrogen Receptor‐Positive
MRI: Magnetic resonance imaging
NDPH: New daily persistent headache
PR +: Progesterone Receptor‐Positive
VAS: visual analog scale

## 1 Introduction

Bone metastases are prevalent in several advanced cancers, most notably in lung, breast, and prostate cancers [1]. The most frequent site of bony metastasis in the axial skeleton is the lumbar spine, whereas the femur is the common site in the appendicular skeleton [2]. Conversely, skull metastasis is rare and frequently identified as an incidental finding [3]. This skull metastasis may be osteolytic or osteosclerotic. The calvaria consist of three distinct layers; the inner table, bone marrow, and the outer table. Calvarial metastasis typically involves all three layers, with hematogenous spread via Batson valveless venous plexus considered the most probable mechanism [4]. Approximately 50% of these patients remain asymptomatic. However, calvarial metastasis can cause focal pain; furthermore, if the lesion invades the dura and intradural space, patients may present with signs and symptoms of elevated intracranial pressure. Breast carcinoma metastasizing to the calvarium and manifesting as headaches or other neurological symptoms is an exceedingly rare phenomenon [3]. As headache is a common complaint, it is often overlooked as a potential symptom of any carcinoma. Calvarial metastasis presenting with new‐onset daily persistent headache (NDPH) is a rare occurrence that poses significant diagnostic and management challenges in patients with breast carcinoma.

NDPH is a subtype of “chronic daily headache” characterized by a new‐onset headache that becomes a daily entity, remaining continuous from onset with mild‐to‐moderate severity, and lasts for more than 3 months [5, 6]. A definitive diagnosis of NDPH is made only after excluding various primary headache syndromes and potential secondary causes. The primary headaches that must be excluded include chronic migraine, chronic tension‐type headache, hemicrania continua, trigeminal autonomic cephalalgia, and hypnic headache [6]. To establish a diagnosis of NDPH, it is also essential to rule out secondary headache etiologies, such as idiopathic intracranial hypertension (characterized by raised intracranial pressure and papilledema), post‐traumatic headache, chronic meningitis (presenting with fever and neck rigidity), and cerebral venous thrombosis (marked by signs of raised intracranial pressure and the characteristic empty delta sign on MRI). This case report details a breast carcinoma survivor who, after 30 months post‐treatment, presented with NDPH. Following a thorough investigation, she was found to have calvarial metastasis presenting as secondary NDPH.

### 1.1 Case Presentation

A 49‐year‐old Indian woman presented to the neurology outpatient clinic in August 2024, with a persistent headache of over 3‐month duration. The headache had an insidious onset, described as continuous, holocranial, and dull aching. There were no aggravating or relieving factors, nor any diurnal or postural variations. The patient rated the pain severity as 8/10 on the visual analog scale (VAS), with no significant relief from over‐the‐counter analgesics. She reported no history of similar complaints in past, trauma, fever, nausea, or vomiting. Her medical history was significant for carcinoma of the left breast, for which she had received treatment two and a half years prior. Upon clinical examination, there were no focal neurological deficits, and neck rigidity was absent. Local examination of the scalp revealed frontal bossing and a 2 × 2 cm midline swelling; it was firm in consistency, immobile, and non‐tender, with ill‐defined margins. The swelling appeared to originate from the underlying bone, with no overlying raised temperature or palpable pulsations. Paranasal sinuses and fundoscopic examination revealed normal findings. An examination of the left chest wall, contralateral breast, bilateral axilla and supraclavicular fossa were all within normal limits.

### 1.2 Significant Past History

In March 2021, the patient was diagnosed with carcinoma of the left breast, clinically staged as cT3N1M0 (stage IIIA) according to American Joint Committee on Cancer (AJCC), eighth edition. A trucut biopsy from the left breast mass revealed invasive ductal carcinoma (IDC) of the breast. Immunohistochemistry (IHC) confirmed that the tumor was estrogen receptor‐positive, progesterone receptor‐positive, and human epidermal growth factor receptor‐negative (ER +, PR +, and Her2‐). Following appropriate baseline investigations that ruled out distant metastasis, the patient received neoadjuvant chemotherapy consisting of three cycles of paclitaxel and carboplatin administered every three weeks. This was followed by a left Patey's mastectomy. Histopathological analysis confirmed grade II, IDC, 3.2 × 2 × 1.8 cm in size, with negative margins and with no evidence of lympho‐vascular invasion and perineural invasion; lymph node 10/12 were positive (ypT2ypN3a). Afterwards, the patient received three cycles of adjuvant chemotherapy. The patient then received external beam radiotherapy (EBRT) to the left chest wall, axilla, and left supraclavicular fossa. Upon completion of EBRT in February 2022, she was started on oral letrozole 2.5 mg daily.

### 1.3 Management

The patient was initially started on oral flunarizine 10 mg daily at bedtime, indomethacin 25 mg three times a day, and a combination of pantoprazole and domperidone (40/10 mg) once daily. She subsequently underwent a gadolinium‐enhanced magnetic resonance imaging (MRI) of the brain, which revealed large (3.6 × 1.8 cm) heterogeneous enhancing areas within the diploic space of the frontal bone (Figure 1). A contrast‐enhanced computed tomography (CECT) of the thorax and abdomen showed no evidence of distant metastasis elsewhere, and routine blood investigations were well within normal limits. The case was discussed with a neurosurgeon regarding an excisional biopsy of the calvarial mass; however, because the lesion was located over the superior sagittal sinus, an excisional biopsy was deemed unfeasible.

**FIGURE 1:**
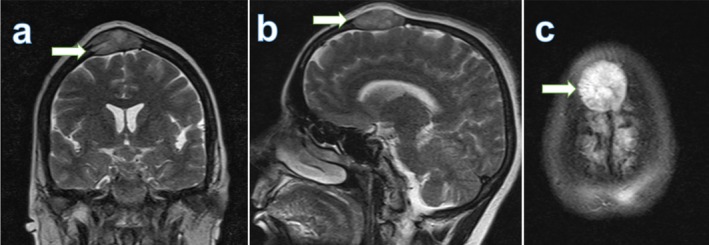
A contrast‐enhanced MRI of the brain reveals a large (3.6 cm × 1.8 cm) heterogeneously enhancing mass in the diploic space of the frontal bone. No calvarial breach was noted, and the underlying brain parenchyma and scalp appeared normal. (A) Coronal T2‐weighted image showing a mass lesion in a parasagittal location, superiorly. The white arrow points to a mass lesion involving the frontal bone. The lesion appears to abut the superior aspect of the brain (B). Sagittal T2‐weighted image demonstrates the extra‐axial nature of the mass (outside the brain parenchyma) and its broad base against the inner table of calvarium (skull bone). The white arrow points to the mass lesion's superficial aspect, which appears to be a bulging scalp slightly. (C) A contrast‐enhanced T1‐weighted image, high axial section, shows a hyperintense near‐central mass lesion (arrow).

The patient received palliative EBRT of dose 30 Gray in 10 fractions to the involved metastatic site, which she tolerated well. Following EBRT, she began a targeted therapy regimen of oral palbociclib (125 mg) for 21 days followed by a 7‐day rest period as part of a 4‐week cycle. Additionally, she received intramuscular fulvestrant (500 mg) injection on day 1 every two weeks for the first three cycles, followed by maintenance doses every four weeks [7]. At the 3‐month follow‐up following EBRT, her pain significantly subsided with VAS score of 2/10. Blood parameters including complete blood count, renal, and liver function tests were within normal range, and clinical examination revealed partial response of the scalp swelling.

## 2 Discussion

The incidence of hormone‐positive (ER +, PR +) breast carcinoma metastasizing to the brain is approximately 14%, most of which is symptomatic [8]. However, breast carcinoma metastasizing specifically to the calvarium, leading to headaches or other neurological symptoms, remains exceedingly rare [3].

Current guidelines from the National Institute for Health and Care Excellence and the American College of Emergency Physicians for adult patients presenting with acute headaches without any systemic symptoms recommend neuro‐radiological imaging, especially in the context of known cancer primary [9, 10]. While CT scans are commonly utilized for the diagnosis of bone lesions. MRI helps in screening for metastases in both skull and the brain. MRI features of calvarial metastases include low‐to‐iso‐intensity signals on T1‐weighted images, and T2‐weighted images show heterogeneous enhancement following gadolinium administration [11]. If a diagnosis of brain metastasis is established via neuroradiological imaging in a known histologically confirmed cancer primary, multimodality treatment is warranted, which includes surgery with or without EBRT or systemic pharmacotherapy and EBRT including whole‐brain radiotherapy with or without boost. Systemic pharmacotherapy alone can be initiated based on prognostic factors (such as number of metastasis, extra CNS disease status, poor performance status, and expected survival less than 3 months) [12, 13]. Palliative EBRT serves as an effective alternative treatment for symptomatic pain caused by skull metastasis. Traditionally, systemic bone metastasis was treated with a low‐dose single fraction of 8 Gray based on RTOG 97–14 results. However, this study also indicated that 3‐year retreatment rates were higher for patients receiving 8 Gray in single fraction compared to those receiving 30 Gray in 10 fractions (15% vs. 5%, *p* = 0.01) [14].

To the best of our knowledge, there are only two case series and one case report available in the literature regarding isolated calvarial metastasis from primary breast carcinoma [3, 15, 16]. Gupta et al., reported 10 cases of calvarial metastasis from various primaries; out of which six were of primary breast carcinoma. Patients received palliative EBRT of dose 20 Gray in five fractions [3]. Similar to the present case, the patient received palliative EBRT. However, a 30 Gray (higher) dose was used for the metastatic site for better long‐term pain control, and along with it, systemic targeted therapy was also used. Miro et al., described a 44‐year‐old lady presented with monocular blindness. MRI showed skull bone metastasis, and the patient was managed with combined systemic chemotherapy and cranial EBRT [16]. Similarly, in the current case, MRI was utilized to diagnose metastasis, and treatment consisted of EBRT and systemic targeted therapy. The various clinical, radiological, and treatment details of the above two studies are compared in Table 1. Kotecha et al., employed radiosurgery to treat calvarial metastasis in five patients with breast carcinoma. They delivered a total dose of 15–24 Gray and achieved a 90% local control rate at the end of 1 year [16]. While the lesion volumes in their case series were small (0.5–6.1 cc), the lesion in the present case was larger (3.6 × 1.8 cm); therefore, EBRT was administered.

**TABLE 1:** Summary of clinical, radiological, and treatment details of previously reported studies of calvarium metastasis in breast cancer patients.

Studies	Age (years) / sex	Breast carcinoma laterality/histopathological diagnosis/stage	Duration between breast cancer and calvarial metastases	Symptom at presentation	Diagnosis (CEMRI brain/CT brain)	Treatment	Adjuvant Therapy	Follow up/status
**Gupta et al., 2022**	67/F	Left/IDC	3 months	Slurring of speech and angle of mouth deviation	Calvarial and extradural metastases with infiltration into brain parenchyma	Palliative WBRT 20 Gray in five fractions	Palliative CTH	NA
	60/F	Right/IDC	5 months	Decreased sensorium, pain, and weakness in the right lower limb	Multiple lytic lesions in calvaria with soft tissue in occipital bone	Palliative WBRT 20 Gray in five fractions		NA
	42/F	Left/IDC	Simultaneously diagnosed	Headache	Multiple calvarial metastases	Palliative WBRT 20 Gray in five fractions		NA
	45/F	Left/IDC	Simultaneously diagnosed	Left facial weakness	Occipital bone metastases	Palliative WBRT 20 Gray in five fractions		NA
	70/F	Right/IDC	Simultaneously diagnosed	Headache	Occipital bone metastases	Palliative EBRT to occipital region		NA
	45/F	Left		Headache, vomiting, generalized weakness, and hypercalcemia	Calvarial metastases without parenchymal involvement	Bisphosphonates and palliative CTH		NA
**Miro et al., 2003**	44/F	Right/IDC	Simultaneously diagnosed	Gradual loss of vision (left monocular blindness)	Extensive lesion of skull compressing left frontal lobe extending to the base of skull and infiltrating left optic nerve	Palliative systemic CTH with WBRT		NA
**Present study**	49/F	Left/IDC/stage IIIA	30 months	Headache	Calvarial lesion involving frontal bone without parenchymal involvement	Palliative EBRT to metastatic site 30 Gray in 10 fractions	Targeted therapy	Three months/alive with disease

*Abbreviations:* CEMRI, contrast‐enhanced magnetic resonance imaging; CT, computed tomography; CTH, chemotherapy; EBRT, external beam radiotherapy; F, female; IDC, invasive ductal carcinoma; NA, not available; WBRT, whole‐brain radiotherapy.

Surgical resection is typically indicated in cases involving neurological deficits, significant bone destruction with dura infiltration, painful masses, or for diagnostic confirmation [17]. Management strategies for breast carcinoma have made significant advancements even in metastatic settings. It is now possible to delay progression of disease and enhance the quality of life of these patients.

## 3 Conclusion

Early neuro‐radiological imaging is mandatory in cancer survivors presenting with atypical headaches in the absence of systemic symptoms. Timely diagnosis of calvarial metastasis is critical in preventing further disease progression and enhancing the patient's quality of life.

## Author Contributions

**Neha Lall:** data curation, formal analysis, validation, visualization, writing – original draft. **Anand Kumar:** conceptualization, data curation, formal analysis, investigation, resources, supervision, validation, writing – review and editing. **Deepika Joshi:** investigation, methodology, supervision, validation, writing – review and editing. **Pramod Kumar Singh:** data curation, investigation, supervision, visualization, writing – review and editing.

## Funding

The authors have nothing to report.

## Ethics Statement

This study did not require ethical approval as per the Institute's ethical policy for single‐case reports. CARE guidelines for case reports were followed.

## Consent

The patient gave written informed consent to publish this case report and accompanying images (Figure 1).

## Conflicts of Interest

All authors declare no competing interests.

## Data Availability

Data sharing not applicable to this article as no datasets were generated or analysed during the current study.
